# *Fusobacterium* is toxic for head and neck squamous cell carcinoma and its presence may determine a better prognosis

**DOI:** 10.1002/cac2.12588

**Published:** 2024-07-06

**Authors:** Anjali Chander, Jacopo Iacovacci, Aize Pellon, Rhadika Kataria, Anita Grigoriadis, John Maher, Cynthia Sears, Gilad Bachrach, Teresa Guerrero Urbano, Mary Lei, Imran Petkar, Anthony Kong, Tony Ng, Ester Orlandi, Nicola Alessandro Iacovelli, Loris De Cecco, Mara Serena Serafini, David Moyes, Tiziana Rancati, Miguel Reis Ferreira

**Affiliations:** ^1^ Centre for Host‐Microbiome Interactions, King's College London London UK; ^2^ Comprehensive Cancer Centre, King's College London London UK; ^3^ Department of Oncology Guys and St Thomas NHS Foundation Trust London UK; ^4^ Data Science Unit Fondazione IRCCS Istituto Nazionale dei Tumori Milan Italy; ^5^ Medicine Department Johns Hopkins University School of Medicine Baltimore Maryland USA; ^6^ Institute of Biomedical and Oral Research, The Hebrew University‐Hadassah School of Dental Medicine, Hadassah Ein Kerem Campus Jerusalem Israel; ^7^ Dipartimento di scienze Clinico‐Chirurgiche, Diagnostiche e Pediatriche Università degli studi di Pavia Pavia Italy; ^8^ Radiation Oncology Department Fondazione IRCCS Istituto Nazionale dei Tumori Milan Italy; ^9^ Department of Medicine Weill Cornell Medicine New York USA

**Abbreviations**
HNSCC: Head and Neck Squamous Cell Carcinoma
RA: relative abundance
OS: Overall Survival
CI: Confidence Interval
DSS: Disease‐Specific Survival
HR: Hazard Ratio
TCMA: The Cancer Microbiome Atlas
TCGA: The Cancer Genome Atlas
PFS: Progression‐Free Survival
HPV: Human Papillomavirus
OSCC: Oral Squamous Cell Carcinoma
MOI: Multiplicity of Infection
LDH: Lactate Dehydrogenase
ROC: Receiver Operating Characteristic
Fnuc: *Fusobacterium nucleatum*
Fper: *Fusobacterium periodonticum*
FnucHI: Group of patients where RA of Fnuc is above the cohort median
FnucLO: Group of patients where RA of Fnuc is below the cohort median
FperHI: Group of patients where RA of Fper is above the cohort median
FperLO: Group of patients where RA of Fper is below the cohort median
ATP: Adenosine triphosphate
16S rRNA: 16S ribosomal RNA

Head and neck squamous cell carcinoma (HNSCC) is a devastating disease. Despite morbid treatment, 5‐year survival rates remain poor (28%‐67%) [1]. There is a significant knowledge gap regarding how the microbiota may impact HNSCC treatment efficacy [2]. We used microbiome data from two independent cohorts to test and validate the hypothesis that oral bacteria are associated with HNSCC prognosis and in vitro models to investigate mechanistic underpinnings. Methods are detailed in Supplementary Materials.

We first explored associations between the relative abundance (RA) of bacterial genera and overall survival (OS) time in 155 patients with mucosal HNSCC available in the Cancer Microbiome Atlas (TCMA, Supplementary Table S1, Supplementary Text). The distribution of bacterial genera is shown in Supplementary Figure S1. Linear stepwise and Cox regression modeling evaluated associations between these genera and OS/DSS. Only *Fusobacterium* detectability was associated with both better OS (hazard ratio [HR] = 0.35, 95% confidence interval [CI] = 0.15‐0.83], *P* = 0.018, Supplementary Figure S2A) and better disease‐specific survival (DSS; 0.28 [0.15‐0.83], *P* = 0.031, Supplementary Figure S2B). Kaplan‐Meier survival analysis mirrored these results (Figure 1A‐B). Additionally, *Fusobacterium* was more abundant in tumors compared to normal tissue (Supplementary Figure S3A‐B), whereas a cognate Gram‐negative oral commensal anaerobe, *Prevotella*, was not (Supplementary Figure S3C‐D). Receiver operating characteristic (ROC) analysis identified a *Fusobacterium* RA cutoff of 0.016 (specificity: 92.7%; sensitivity: 28.8%). Patients with RA above the threshold had better OS and DSS (Supplementary Figure S4).

**FIGURE 1:**
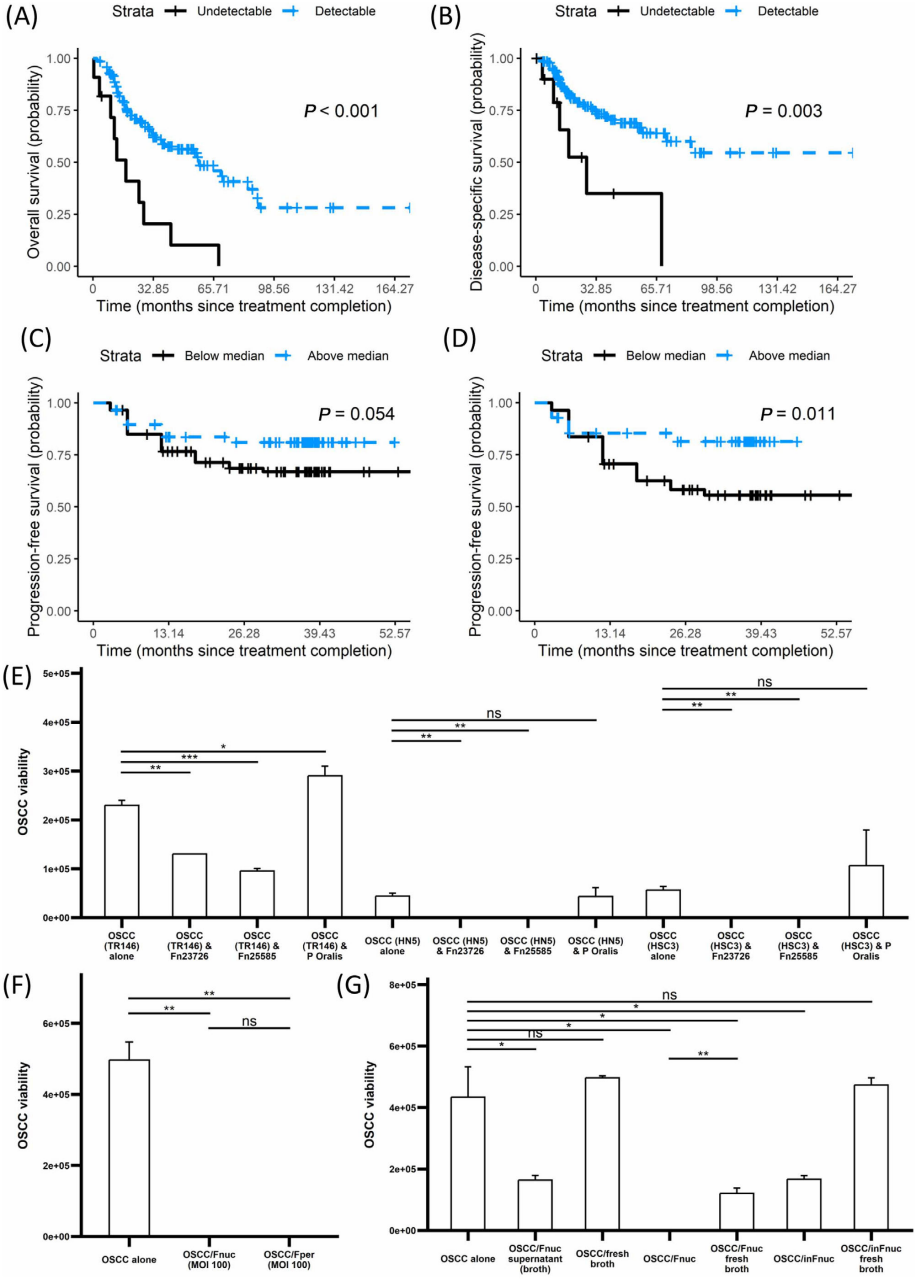
*Fusobacterium* detectability enhances survival in HNSCC, while its presence in co‐culture is toxic for HNSCC. In the TCMA cohort, intra‐tumoral *Fusobacterium* detectability determines better OS (A) and DSS (B). In the MicroLearner cohort, a salivary *Fusobacterium* RA above the cohort median determines better PFS in the full cohort (C) and in the HPV‐neg cohort (D). (E) In 2D co‐culture, OSCC toxicity with *F. nucleatum* (MOI 1) and lack thereof with *P. oralis* (NCTC 11459, MOI 1) is reproducible in the TR146, HN5 and HSC3 cell lines. Additionally, it is reproducible with different *F. nucleatum* strains (ATCC 23726, ATCC 25586; MOI 1). (F) *F. nucleatum* and *F. periodonticum* cause OSCC killing at similar magnitude. (G) OSCC viability in single culture with or without the supernatant of *Fnuc* culture, or fresh broth (i.e., broth without *Fnuc* supernatant), or *F. nucleatum* (either alive (Fnuc) or heat‐inactivated (inFnuc)), showing that *Fnuc* supernatant and/or live *Fnuc* (i.e., producing supernatant molecules) are sufficient and necessary for OSCC toxicity. Significance levels: ns: not significant; *: *P* <0.05; **: *P <* 0.01; ***: *P <* 0.001; ****: *P <* 0.0001; ns: ###. Abbreviations: DSS, disease‐specific survival; Fnuc, *F. nucleatum*; Fper, *F. periodonticum*; HNSCC, head and neck squamous cell carcinoma; inFnuc, heat‐inactivated *F. nucleatum*; MOI, multiplicity of infection; OS, overall survival; OSCC, oral squamous cell carcinoma; PFS, progression‐free survival.

Next, we questioned whether any particular *Fusobacterium* species were associated with survival. Patients were stratified into groups with detectable and undetectable species (Supplementary Figure S5). In Cox regression, only *Fusobacterium nucleatum* detectability was significantly associated with OS (HR: 0.43 [95% CI: 0.19‐0.97], *p* = 0.042; Supplementary Figure S6). Kaplan‐Meier modeling showed that *F. nucleatum* detectability was associated with improved OS (*P* <0.001, Supplementary Figure S7A), with a trend for improved DSS (*P* = 0.096, Supplementary Figure S7B).

In multivariate Cox modeling with established predictors of survival (disease stage, smoking and Human Papilloma Virus [HPV] status), both *Fusobacterium* and *F. nucleatum* detectability were strongly associated with OS (*P* < 0.001 for both, Supplementary Figures S8A/S9A) and DSS (*P* < 0.001 and *P* = 0.015 for each respectively, Supplementary Figures S8B/S9B).

To test the validity of these results, we evaluated whether the abundance of *Fusobacterium* was also predictive of treatment efficacy in the separate MicroLearner cohort (*n* = 175; described in Supplementary Text and Supplementary Table S2) by dividing it into patient groups with *Fusobacterrium* RA either below (FusoLO) or above (FusoHI) the cohort median, as the commensal nature of *Fusobacterium* in the oral cavity makes detectability nearly universal in saliva [3]. We used progression‐free survival (PFS) as an endpoint because, with a median follow‐up of 33.6 months (range 4‐57 months), very few deaths (*n* = 6, 3.4%) had occurred. FusoHI patients had a trend for better PFS (*P* = 0.054; Figure 1C). Only 10 events (15.6%) of progression were observed in patients with HPV‐positive oropharyngeal cancer, so we conducted a separate analysis including all patients except these (“HPVneg cohort”; *n* = 111, 29.7% event rate), where FusoHI patients had significantly better PFS (*P* = 0.011, Figure 1D). ROC analysis identified a salivary *Fusobacterium* RA cutoff of 2.760 in this cohort (specificity: 65.2%; sensitivity: 55.8%). Patients with RA above this threshold had better PFS (Supplementary Figure S10). *F. nucleatum* (Fnuc) and *F. periodonticum* (Fper) were the most abundant fusobacterial species. There was a non‐significant trend for better PFS in FnucHI (*F. nucleatum* RA > median *F. nucleatum* RA) patients, but FperHI patients had significantly better PFS (*P* = 0.021; Supplementary Figure S11).

Given our clinical observations, we reasoned that *Fusobacterium* may contribute to HNSCC killing. We initially explored the effect of *F. nucleatum* on oral SCC (OSCC) evaluated with an ATP‐based viability assay. TR146 cells were infected with *F. nucleatum* at multiplicity of infection (MOI) ranging from 0.5 to 5. With increasing MOI, a more significant reduction in OSCC cell viability was observed (Supplementary Figure S12A). Separate experiments using lactate dehydrogenase (LDH) activity and crystal violet assays validated these findings (Supplementary Figure S12B‐C). We also tested whether the *F. nucleatum* medium caused any OSCC death if added without any previous contact with bacteria and confirmed that it did not (Supplementary Figure S12D). A significant decrease in viability was observed from 24h post‐infection (Supplementary Figure S13).

To test whether the observed effects of *F. nucleatum* on OSCC cytotoxicity were strain‐specific, cell‐line specific and not a general characteristic of oral commensal anaerobes, we co‐cultured multiple cell lines of OSCC (TR146, HN5 and HSC‐3) with either of two *F. nucleatum* strains or *Prevotella oralis* (MOI = 100) and evaluated their effect on OSCC viability (Figure 1E), validated with a crystal violet assay (Supplementary Figure S14). *P. oralis*, like *F. nucleatum*, is an oral commensal Gram‐negative anaerobe. *P. oralis* infection did not impact OSCC viability, while both *F. nucleatum* strains caused a reduction in OSCC viability. We next questioned whether other *Fusobacterium* species caused OSCC killing. We tested the effect of *F. periodonticum* on OSCC cultures at MOI 100 and found that it caused OSCC killing similarly to *F. nucleatum* (Figure 1F). At lower MOI (0.5‐5), OSCC killing was also overall similar between the two species and rose with MOI (Supplementary Figure S15). These results suggest that other *Fusobacterium* species which are phylogenetically close to *F. nucleatum*, but not all oral commensal Gram‐negative anaerobes can cause OSCC killing.

We next asked whether OSCC killing was mediated by a surface protein or by secreted compounds/metabolites (Figure 1G). Firstly, OSCC cells were infected with *F. nucleatum*, which was either alive or heat‐inactivated (in*Fnuc*), and OSCC viability was assessed. We also tested whether the supernatant of *F. nucleatum* culture was sufficient to cause OSCC death. *F. nucleatum* supernatant caused OSCC killing, whereas fresh medium did not. Co‐culture of *F. nucleatum* washed in fresh broth significantly attenuated OSCC killing compared to growth broth, suggesting continued production of supernatant in co‐culture. in*Fnuc* caused OSCC killing only when added to co‐culture with growth broth but not with fresh broth. Separately, we used transwell inserts to prevent direct contact of *F. nucleatum* with OSCC while allowing for any secreted molecules to move freely between them (Supplementary Figure S16). Significant cell killing was observed in transwell replicates, more substantially when *F. nucleatum* was in direct contact with OSCC, which may be attributable to higher local concentrations in direct contact co‐culture compared to transwell replicates. Taken together, these results indicate that *F. nucleatum* mediates OSCC killing primarily via the bacterial secretome.

Although colorectal cancer studies indicate that *F. nucleatum* contributes to oncoprogression and treatment resistance, these bacteria are not common constituents of the normal intestinal microbiota, whereas they are common components of the normal oral microbiota [4]. Previous studies often assume that a higher tumoral abundance of *Fusobacterium*, which we also detected, indicates its oncogenic role [5]. However, our findings suggest that its presence may enhance HNSCC treatment efficacy. Limitations of this study are discussed in the Supplementary Text.

In summary, our preliminary research suggests that *Fusobacterium* actively determines survival outcomes in HNSCC. Ongoing research will validate its role as a predictive biomarker in HNSCC and dissect the mechanism by which fusobacteria cause HNSCC killing.

## AUTHOR CONTRIBUTIONS

Miguel Reis Ferreira conceived and designed the study. Miguel Reis Ferreira, Anjali Chander, Aize Pellon and David Moyes designed the experiments. Jacopo Iacovacci and Tiziana Rancati designed and analyzed MicroLearner study data. Miguel Reis Ferreira, Anjali Chander and Jacopo Iacovacci analyzed the data. Jacopo Iacovacci, Rhadika Kataria, Anita Grigoriadis, David Moyes and Tiziana Rancati supported data analysis. Anjali Chander, Jacopo Iacovacci, Tiziana Rancati and Miguel Reis Ferreira reviewed the results, interpreted the data and wrote the manuscript. Anjali Chander, Jacopo Iacovacci, Aize Pellon, Rhadika Kataria, Anita Grigoriadis, John Maher, Cynthia Sears, Gilad Bachrach, Teresa Guerrero Urbano, Mary Lei, Imran Petkar, Anthony Kong, Tony Ng, Ester Orlandi, Nicola Alessandro Iacovelli, Loris De Cecco, Mara Serena Serafini, David Moyes, Tiziana Rancati and Miguel Reis Ferreira critically reviewed the manuscript for important intellectual content and approved the final version. Miguel Reis Ferreira has primary responsibility for the final content of the manuscript. All authors reviewed and approved the final manuscript for submission.

## CONFLICT OF INTEREST STATEMENT

The authors declare no competing interests.

## FUNDING INFORMATION

Wilson + Olegario: Philanthropy through Guys Cancer Charity (MRF) Guys Cancer Charity (MRF) Cancer Research UK through the City of London Cancer Centre (MRF) Fondazione Regionale per la Ricerca Biomedica, grant ID 2721017 (JI).

## ETHICS APPROVAL AND CONSENT TO PARTICIPATE

The MicroLearner observational study of the microbiome in patients treated with radiotherapy for head and neck and prostate cancers was registered on ClinicalTrials.gov (ID: NCT03294122) and approved by the local Ethical Committee (ID INT 11/17). All patients provided written informed consent and agreed that incidental findings would not be disclosed to them or any clinician.

## Supporting information

Supporting information

## Data Availability

Data from the Cancer Microbiome and Cancer Genome Atlases are publicly available, as indicated in the text. All other data generated or analyzed during this study are included with the published article and its supplementary information files.
