# Supplementary material for: Fusobacterium is toxic for head and neck squamous cell carcinoma and its presence may determine a better prognosis
Source: Cancer Commun (Lond). 2024 Jul 6;44(8):879–83. doi: 10.1002/cac2.12588 (PMC11337922; doi:10.1002/cac2.12588)
Supplement: Supplementary file 1 — Supporting information [file CAC2-44-879-s001.docx]

**Supplementary Files for**

*Fusobacterium* is toxic for head and neck squamous cell carcinoma and its presence may determine a better prognosis

Anjali Chander,1,2,3; Jacopo Iacovacci,4; Aize Pellon,1; Rhadika Kataria,2; Anita Grigoriadis,2; John Maher,2; Cynthia Sears,5; Gilad Bachrach,6; Teresa Guerrero Urbano,2,3; Mary Lei,2,3; Imran Petkar,2,3; Anthony Kong,2,3; Tony Ng,2; Ester Orlandi,7; Nicola Alessandro Iacovelli,8; Loris De Cecco,8; Mara Serena Serafini,8,9; David Moyes,1; Tiziana Rancati,4*; Miguel Reis Ferreira,1,2,3*

**Affiliations:**

1: Centre for Host-Microbiome Interactions, King’s College London, London, SE1 1UL, United Kingdom

2: Comprehensive Cancer Centre, King’s College London, London, SE1 1UL, United Kingdom

3: Department of Oncology, Guys and St Thomas NHS Foundation Trust, London, SE1 3SS, United Kingdom

4: Data Science Unit, Fondazione IRCCS Istituto Nazionale dei Tumori, Milan, 20133, Italy

5: Medicine Department, Johns Hopkins University School of Medicine, Baltimore, 21287, Maryland, United States of America

6: Institute of Biomedical and Oral Research, The Hebrew University-Hadassah School of Dental Medicine, Hadassah Ein Kerem Campus, Jerusalem, 91120, Israel.

7: Dipartimento di scienze Clinico-Chirurgiche, Diagnostiche e Pediatriche, Università degli studi di Pavia, Pavia, 19 27100, Italy

8: Radiation Oncology Department, Fondazione IRCCS Istituto Nazionale dei Tumori, Milan, 20133, Italy.

9: Department of Medicine, Weill Cornell Medicine, New York, 10021, United States of America

*Corresponding authors:

**1 - Miguel Reis Ferreira**

Affiliations: Centre for Host-Microbiome Interactions, King’s College London, London, SE1 1UL, United Kingdom; Comprehensive Cancer Centre, King’s College London, London, SE1 1UL, United Kingdom; Department of Oncology, Guys and St Thomas NHS Foundation Trust, London, SE1 3SS, United Kingdom

E-mail: [Miguel.ReisFerreira@kcl.ac.uk](mailto:Miguel.ReisFerreira@kcl.ac.uk)

**2 - Tiziana Rancati**

Affiliations: Data Science Unit, Fondazione IRCCS Istituto Nazionale dei Tumori, Milan, 20133, Italy

E-mail: [tiziana.rancati@istitutotumori.mi.it](mailto:tiziana.rancati@istitutotumori.mi.it)

# **Supplementary Materials and Methods**

**1. The Cancer Microbiome Atlas (TCMA) data source and processing**

Microbiome data (genus and species levels relative abundance tables) of head and neck squamous cell carcinoma (HNSCC) patients were obtained from the Cancer Microbiome Atlas (TCMA) database (<https://tcma.pratt.duke.edu>, last accessed August 2023). Details about the microbiome detection pipeline (PathSeq) used to derive this data are made available by the authors of the resource in its accompanying publication [1].

Taxa where relative abundances were 0 in all patients were removed from the analysis. Metadata, including curated survival data, was obtained from the TCMA and The Cancer Genome Atlas (TCGA) databases, the latter through the UCSC cancer genome browser ((<https://tcga.xenahubs.net>). The outcomes assessed were overall (OS) and disease-specific (DSS) survival. Only patients where microbiome and survival outcomes were available were included in the analysis, with *n* = 155 patients retained. Of these, 21 patients also had matched normal tissue microbiome data.

To explore associations between the microbiome and survival, forward and backward linear stepwise regression models were computed, including all genera with a relative abundance (RA) of > 0 in at ≥1 patient. The forward stepwise model was best fit to the data (Akaike Information Criterion 2,504 vs. 2,573); therefore, genera detectable in at least 10% of patients (as less prevalence was deemed to be clinically irrelevant) and where RA was significantly associated with OS time in this model were carried over for further analysis. These genera, as well as the results of forward stepwise modeling and their detectability (expressed in percentage of patients with relative abundance (RA) > 0) are detailed in **Supplementary Table S3**. Cox regression was used to evaluate associations between these genera and OS/DSS. Patients were stratified into groups with undetectable (RA = 0) or detectable (RA > 0) bacterial genera. Cox regression was used to evaluate associations between these genera and OS/DSS with patients stratified into groups with undetectable (RA = 0) or detectable (RA > 0) bacterial genera.

**2. MicroLearner study**

The MicroLearner observational study of the microbiome in patients treated with radiotherapy for head and neck and prostate cancers was registered on ClinicalTrials.gov (ID: NCT03294122) and approved by the Ethics Committee of the Fondazione IRCCS Istituto Nazionale dei Tumori (ID INT 11/17). All patients provided written informed consent and agreed that incidental findings would not be disclosed to them or any clinician.

The trial enrolled patients with HN-SCC between February 2017 and February 2020. All patients were treated with high-dose radiotherapy with curative intent (either primary or post-operative radiotherapy) at the Fondazione IRCCS Istituto Nazionale dei Tumori in Milan (Italy). Detailed information on patient- and treatment-related features were prospectively registered using standardized Case Report Forms (CRFs). Information on local/regional/distant relapse was prospectively collected at the end of treatment, at 3 and 6 months after treatment completion and then every 6 months until five years post-treatment. Cohort details are reported in **Supplementary Table S2**.

***Radiotherapy Treatment***

All patients received Volumetric Modulated Arc radiotherapy with photons X≥6MV and daily control of the set up using cone-beam computed tomography (CT) according to institutional standards. Radiotherapy was delivered with a simultaneous integrated boost at 2-2.2Gy/fraction. Total prescription doses in the radical definitive setting were 66-70.95Gy to the high-dose target, and 57.6-66Gy and 54-59.5Gy to the intermediate and low-dose targets, respectively. In the post-surgery setting, patients received 60-70.95Gy to the high-dose target, and 54-66Gy and 58.6-59.4Gy to the intermediate and low-dose targets. Irradiation targets and constraints to organs at risk followed international guidelines and institutional protocols. Patients were allowed to receive induction chemotherapy or concomitant chemotherapy at the clinician's discretion.

***Saliva Sample Collection and Processing***

Salivary samples were collected using the OMNIgene•ORAL kit (DNA Genotek Inc. Ottawa, ON, Canada) and stored at +4°C before being delivered to the centralized laboratory for bacterial community characterization analyses.  On arrival, DNA was extracted by QIAsymphony DSP Virus /Pathogen Midi kit (Qiagen, Hilden, Germany) after mechanical lysis with silica beads and processed on a QIAsymphony station (Qiagen, Hilden, Germany). Microbial DNA was quantified using a Qubit fluorometer (ThermoFisher, Waltham, Massachusetts, United States of America), and quality was assessed using a 4200 TapeStation (Agilent, Santa Clara, California, United States of America). All samples used for bacterial community profiling had DNA integrity number >7.   The NGS libraries were constructed using the 16S Metagenomics kit (ThermoFisher, Waltham, Massachusetts, United States of America) following the manufacturer's instructions. The 16S rRNA gene was amplified with primer sets recognizing V2, V3, V4, V6-7, V8 and V9 hypervariable regions in 2 separate PCR reactions. Fifty ng of amplicons were combined and processed for library prep using Ion Plus Fragment Library Kit and Ion Xpress Barcodes Adapters (ThermoFisher). After PCR amplification (1 cycle of 95°C for 5 min; 5 cycles of 95°C for 15 s, 58°C for 15 s, 70°C for 1 min) and purification using 1.4 volumes of Agencourt AMPure beads (Beckman Coulter), libraries were eluted and their size and quantity were assessed with TapeStation.  Sequencing was performed for 16S rRNA gene libraries by Ion S5 XL, whereas base calling and demultiplexing were performed by Torrent Suite (ThermoFisher, Waltham, Massachusetts, United States of America).  The ThermoFisher Ion Reporter Software 16S rRNA gene sequence analysis pipeline was used to generate operational taxonomic unit abundances from the 16S rRNA gene reads and to assign taxonomy at the genus and species level.

**3. Mammalian cell culture**

Adherent oral squamous cell carcinoma (OSCC) cell lines were grown in adequate cell culture medium: TR146 cell line, Dulbecco's Modified Eagle's Medium (DMEM):F12 supplemented with 10% fetal bovine serum (FBS) and 1% Penicillin/Streptomycin; HSC3 and HN5 cell lines, DMEM supplemented with 1 mM sodium pyruvate, 10% FBS and 1% Penicillin/Streptomycin; DOK cell line, DMEM (with L-glutamine) supplemented with 5 µg/ml Hydrocortisone, L-glutamine, 10% FBS and 1% Penicillin/Streptomycin.

Non-adherent natural killer (NK) cells (KHYG-1 cell line) were maintained in Roswell Park Memorial Institute (RPMI) cell culture medium supplemented with 20% FBS, 1% L-glutamine, 1% HEPES solution, and 1% Penicillin/Streptomycin, and interleukin-2 (450 U/mL).

All cell lines were incubated at 37^o^C with 5% CO2 and 95% relative humidity. When needed, cells were harvested, either via trypsinization (for adherent cells) or centrifugation (400 g, 5 min), counted using a Neubauer chamber, and plated to perform experiments.

**4. Bacterial culture**

*Fusobacterium nucleatum* (ATCC 23726, 25586), *Fusobacterium periodonticum* (ATCC 33693) and *Prevotella oralis* (NCTC 11459) were grown in fastidious anaerobe agar (FAA) supplemented with 5% defibrinated horse blood in an anaerobic workstation (Don Whitley MACS MG1000, atmosphere composition: 80% Nitrogen, 10% Carbon dioxide and 10% Hydrogen) at 37^o^C. *F. nucleatum* cultures were confirmed by 16S rRNA gene sequencing (following bacterial DNA extraction, purification, and qPCR). To obtain bacteria for experiments, *F. nucleatum* was grown in Brain Heart Infusion (BHI) broth and *P. oralis* in BHI supplemented with 0.1% mucin, 10% FBS and 0.001% vitamin K. Once the anaerobic bacteria had grown visible colonies on FAA-blood agar plates (after 48-72 hours, approximately), one filled plastic loop of the colonies was transferred to reduced BHI medium. The inoculated broth was subsequently returned to the anaerobic workstation to allow bacterial cell growth for 24 hours before use (until the logarithmic growth phase as determined by the calculated growth curve). Quantification of bacteria in cell suspensions was carried out using homemade curves comparing culture optical density at 600 nm (OD600) and the number of colony-forming units.

Where heat inactivation was used, *F. nucleatum* was heat-inactivated by heating the bacterial suspension to 70^o^C in a water bath for 30 min before allowing to fully cool to 37^o^C before infecting OSCC cells. Inactivation of *F. nucleatum* was confirmed by plating 200 µl of the suspension onto fastidious anaerobe agar (with 5% horse blood) plates and observing for bacterial colony growth.

*F. nucleatum* supernatant was prepared by centrifuging the bacterial suspension at 2000G for 10 min. The supernatant was removed and passed through a 0.2µM filter.

**5. Co-culture cell preparation**

One day before co-culture experiments, 2 × 10^5^ OSCC cells were transferred to a transparent 48-well plate in fresh RPMI (supplemented with 20% FBS, 10% L-glutamine and 10% HEPES solution, without penicillin/streptomycin). Where NK cells were used in co-cultures, they were transferred to the 48-well plate (the quantity depending on the KHYG-1 to OSCC ratio to be used) on the day of infection (day 0). For bacteria, after carrying out the steps above (see *'Gram-negative anaerobe preparation*'*),* the OD_600_ of the inoculated broth was measured and using *F. nucleatum/P. oralis* growth curves, the volume of inoculated broth required was calculated (depending on the multiplicity of infection (MOI) required) and then added to the co-cultures. Plates were then transferred to the incubator. In transwell insert experiments, 5x10^5^ OSCC (TR146) cells within 1ml of RPMI medium were plated into each of the wells of a 12-well plate. *F. nucleatum* was added to a 12mm diameter, 0.4μm polycarbonate membrane transwell permeable insert and placed into the wells.

**6. Assays**

We previously reported the development of the aforementioned 2D cell culture model aimed at understanding the contribution of NK cells to OSCC radiotherapy response and how bacteria impacted that relationship [2]. To do so, an ATP-based cell viability assay (CellTiter-Glo 2.0, discussed below) was optimized following previously published principles [2]. Importantly, readouts were measured at 4-6 days post-stimulus (**Supplementary Figure S17**) as radiotherapy responses require a lag time prior to measurable results in cell cultures. Although radiation effects are not described in this paper, this model was used for experiments herein described and differs from previous approaches in microbiology applied to cancer, where readouts are obtained between 4 and 24 hours after stimulus (in this case, bacterial infection of cancer cultures) [3, 4].

***Cell viability assay***

Cell viability was assessed using CellTiter-Glo 2.0® (Promega, Fitchburg, Wisconsin, United States of America) according to the manufacturer's protocol.

To assess viability, the culture medium was aspirated from each of the wells, which were washed 9 times with 300μl warmed PBS to remove the KHYG-1 cells and *F. nucleatum/P. oralis*. 100μl of CellTiter-Glo 2.0® Reagent was added to each of the wells to be tested. Plates were transferred to an orbital shaker for 10 minutes to induce cell lysis, after which the 200μl supernatant was transferred to an opaque white-walled 96-well plate. Luminescence was recorded using manufacturer instrument settings. An integration time of 0.25-1 s per well were used as a guideline.

***LDH assay***

To assess cellular damage, LDH activity in the supernatant, was measured using the Cytotox 96 assay (Promega, Fitchburg, Wisconsin, United States of America) according to the manufacturer's protocol.

50µl of samples were added to the 96-well plate (5µl of culture supernatant and 45µl of PBS). 50µl of the substrate mix was added to each well and the plate was stored in the dark for 30 min at room temperature. 50µl of stop solution (1M acetic acid, stored at 4^o^C) were added to each of the wells and absorbance was measured with microplate reader at 490nm.

***Crystal violet assay***

Cell preparation was carried out as above (excluding cell viability assay), but cells were plated in a 6-well plate. 1 x 10^6^ OSCC cells were plated in each well the day before infection in 1ml of RPMI and IL-2 (without penicillin/streptomycin). On day 0, NK cells and/or *F. nucleatum/P. oralis* were added. After 5 days of incubation, the culture medium was aspirated from the wells and cells were washed 9 times with 1ml warm PBS to remove KHYG-1 cells and/or *F. nucleatum/P. oralis*. One ml of methanol was added to each well and left to fix for 15 min.

Methanol was removed and cells were left to dry within the fume hood on air for 45 min. After fixation, 1ml of 1% crystal violet solution was added to each well and left to incubate for 20 min at room temperature. Wells were washed twice with running water and 1ml 33% acetic acid was added to each of the wells to allow the violet crystals to dissolve. 100µl of solubilized crystal solution from each well was transferred into a transparent 96-well plate and optical density was measured in a microplate reader at 620nm.

**7. Statistical considerations**

All analyses were conducted in R v4.1.2 using the survival, survminer, ggplot2, dplyer and rstatix packages. Statistical significance was defined at *P* ≤ 0.05.

***TCGA and Microlearner data***

We computed descriptive statistics, including means, medians and frequencies, to describe the patient cohort. Forward and backward stepwise linear regression models were applied using the step() function in R with OS time as the dependent variable and relative bacterial abundances of all bacteria (where relative abundances were >0 in at least 1 patient) as explanatory variables. The suitability of each model was evaluated using the Akaike information criterion (AIC), and the model with the lowest AIC was chosen to identify bacterial taxa associated with post-treatment survival.

Bacteria with relative abundance associated with survival in the model with the lowest AIC were then carried over for survival analysis. Cox proportional hazards regression analysis was used to assess the effect of bacterial relative abundance on OS time. Where significance was observed, treatment efficacy was assessed using Kaplan-Meier methods to calculate overall and disease-specific survival from the start of treatment. Patient groups were divided into those where bacteria were detectable vs undetectable (defined as relative abundance = 0), or according to a relative abundance cut-off defined by receiver operating characteristic (ROC) analysis performed using the 'pROC' package in R. Patients were censored at the date they were last known to be alive. Significance was assessed with the log-rank test.

Paired sample comparisons were made using the Wilcoxon signed-rank test. Statistical significance was defined at p≤0.05.

***Cell culture model data***

All experiments were carried out in triplicate. Experiments with different assays and/or conditions were carried out independently. Paired comparisons were made using the paired Student's t test.

All tests were two-sided.

**Supplementary Text**

# **The Cancer Microbiome Atlas (TCMA) cohort**

One hundred fifty-five patients with mucosal HN-SCC were available for analysis. Demographics are summarised in **Supplementary Table S1**. Most (72.9%) patients were male with the median age being 60 years (range: 19-90). The most frequent primary site of HN-SCC was the oral cavity (67.7%), followed by the oropharynx (16.8%). The majority of patients had locoregionally advanced tumors, with 72.9% staged III-IVA. Most patients were treated with surgery (89.0%) and/or radiotherapy (50.3%). The majority of patients were non-smokers or ex-smokers (69.7%) and active drinkers (71.6%).

Median follow-up was 26 months. Median overall survival (OS) of the whole cohort was 57 months and 73 patients died. Median disease-specific survival (DSS) was not reached, with 47 tumor recurrences (14 locoregional, 8 metastatic, data unavailable for 25 patients).

Tumor microbiomes were dominated by 20 genera, with other bacteria accounting for a mean 9% (SD=8%) relative abundance (**Supplementary Figure S2**).

# **The MicroLearner study cohort**

The MicroLearner study (NCT03294122) tested whether salivary microbiota, assessed using 16S rRNA gene sequencing (16Sseq), influence radiation-induced toxicity in patients treated with radiotherapy for HN-SCC. Pre-treatment salivary microbiome was profiled in 175 patients with annotated tumor histology and subsite information. Additionally, MicroLearner is the largest purposefully prospectively-collected microbiome dataset with associated efficacy data in HN-SCC. Unlike the TCMA cohort, the MicroLearner cohort was dominated by patients treated for oropharyngeal and nasopharyngeal cancers (**Supplementary Table S2**).

# **Study limitations**

We acknowledge limitations. Firstly, the tumor microenvironment and its associated microbiota are difficult to replicate within a 2D co-culture model. Neither the TCMA nor MicroLearner cohorts accounted for antibiotic uptake. Recent controversies surrounding the TCGA-derived Poore et al. microbiome database are acknowledged[5]. However, we used TCMA data, which uses a more stringent methodology for bacterial read detection. Also, it was validated by comparing the taxonomy obtained from bioinformatic analysis of whole genome sequencing with the taxonomy obtained from the same samples using de novo 16S rRNA gene sequencing. We acknowledge limitations inherent in using RA, particularly its inability to provide absolute bacterial load information. However, we focused on comparative microbial community analysis rather than quantifying bacterial biomass, so RA offers the most informative and pragmatic approach as it normalizes data and allows for more accurate analysis across cohorts. We also recognize that sampling methods differed between cohorts, (TCMA/biopsies vs MicroLearner/saliva). Our findings suggest that, at the genus level, results are similar across sample types, so salivary sampling may facilitate larger-scale studies. Finally, further mechanistic dissection will require more sophisticated in vitro and animal models.

**Supplementary references**

1. Dohlman AB, Arguijo Mendoza D, Ding S, Gao M, Dressman H, Iliev ID, et al. The cancer microbiome atlas: a pan-cancer comparative analysis to distinguish tissue-resident microbiota from contaminants. Cell Host Microbe. 2021 Feb 10;29(2):281-298.e5.

2. Kleijn A, Kloezeman JJ, Balvers RK, Kaaij M van der, Dirven CMF, Leenstra S, et al. A Systematic Comparison Identifies an ATP-Based Viability Assay as Most Suitable Read-Out for Drug Screening in Glioma Stem-Like Cells. Stem Cells Int. 2016;2016:5623235.

3. Michikawa C, Gopalakrishnan V, Harrandah AM, Karpinets T V, Garg RR, Chu RA, et al. Fusobacterium is enriched in oral cancer and promotes induction of programmed death-ligand 1 (PD-L1). Neoplasia. 2022 Sep;31:100813.

4. Gur C, Ibrahim Y, Isaacson B, Yamin R, Abed J, Gamliel M, et al. Binding of the Fap2 protein of fusobacterium nucleatum to human inhibitory receptor TIGIT protects tumors from immune cell attack. Immunity. 2015 Feb 17;42(2):344-355.

5. Gihawi A, Ge Y, Lu J, Puiu D, Xu A, Cooper CS, et al. Major data analysis errors invalidate cancer microbiome findings. mBio. 2023 Oct 31;14(5):e0160723.

**Supplementary Tables**

# **Supplementary Table S1. Patient demographics (TCMA cohort)**

| Characteristics | | Total (*n*=155) |
| --- | --- | --- |
| Gender: % (*n*) | | |
|  | Male | 72.9% (113) |
|  | Female | 27.1% (42) |
| Age: mean (SD) | | 60 (12.6) |
| Primary site: % (*n*) | | |
| Oral cavity | All oral cavity | 67.7% (105) |
|  | Alveolar Ridge | 3.9% (6) |
|  | Buccal Mucosa | 2.6% (4) |
|  | Floor of mouth | 7.1% (11) |
|  | Hard palate | 1.9% (3) |
|  | Lip | 0.6% (1) |
|  | Oral cavity, NOS | 16.1% (25) |
|  | Oral Tongue | 35.5% (55) |
| Oropharynx | All oropharynx | 16.8% (26) |
|  | Tonsil | 11.0% (17) |
|  | Base of tongue | 5.2% (8) |
|  | Oropharynx, NOS | 0.6% (1) |
| Larynx |  | 14.2% (22) |
| Hypopharynx |  | 1.3% (2) |
| Stage | | |
|  | I | 2.6% (4) |
|  | II | 21.3% (33) |
|  | III | 20.0% (31) |
|  | IVA | 52.9% (82) |
|  | IVB | 1.9% (3) |
|  | IVC | 0.6% (1) |
|  | Stage data NA | 0.6% (1) |
| Treatment: % (*n*) | | |
| Surgery | Received | 89.0% (138) |
|  | Surgery data NA | 11.0% (17) |
| Radiotherapy | Received | 50.3% (78) |
|  | Not received | 34.2% (53) |
|  | Radiotherapy data NA | 15.5% (24) |
| Drinking and smoking status % (*n*) | | |
| Lifelong non-smoker | | 23.2% (36) |
| Ex-smoker | | 46.5% (72) |
| Current smoker | | 29.0% (45) |
| Smoking data NA | | 1.3% (2) |
| Drinker | | 71.6% (111) |
| Non-drinker | | 25.8% (40) |
| Drinking data NA | | 2.6% (4) |

Abbreviations: NA: not available. SD: standard deviation. NOS: Not otherwise specified.

# **Supplementary Table S2. Demographics of the MicroLearner cohort**

| Characteristics | | Total (n=175) |
| --- | --- | --- |
| Gender: % (*n*) | | |
|  | Male | 73.1% (128) |
|  | Female | 26.9% (47) |
| Age: mean (SD) | | 58 (12.0) |
| Primary site: % (*n*) | | |
| Oral cavity |  | 10.3% (18) |
| Oropharynx |  | 44.0% (77) |
| Larynx |  | 8.0% (14) |
| Hypopharynx |  | 1.7% (3) |
| Nasopharynx |  | 27.4% (48) |
| Paranasal sinuses |  | 4.0% (7) |
| Nasal cavity |  | 0.6% (1) |
| Salivary gland |  | 3.4% (6) |
| Lacrimal gland |  | 0.6% (1) |
| Stage: % (*n*) | | |
|  | I | 3.4% (6) |
|  | II | 9.1% (16) |
|  | III | 26.9% (47) |
|  | IVA | 42.3% (74) |
|  | IVB | 15.4% (27) |
|  | IVC | 0.0% (0) |
|  | Stage data NA | 2.9% (5) |
| Treatment: % (*n*) | | |
| Surgery | Received | 37.1% (65) |
|  | Not received | 62.9% (110) |
| Radiotherapy | Received | 100.0% (175) |
|  | Not received | 0.0% (0) |
| Drinking and smoking status: % (*n*) | | |
| Lifelong non-smoker | | 34.9% (61) |
| Ex-smoker | | 56.0% (98) |
| Current smoker | | 9.1% (16) |
| Smoking data NA | | 0.0% (0) |
| Drinker | | 29.1% (51) |
| Ex-drinker | | 5.7% (10) |
| Lifelong non-drinker | | 61.1% (107) |
| Drinking data NA | | 4.0% (7) |

Abbreviations: NA: not available. SD: standard deviation. NOS: Not otherwise specified.

# **Supplementary Table S3. Results of the forward stepwise regression model**

| Bacteria (genus) | Coefficient estimate | Standard error | *t* value | *P* value | Detectability (% of patients) |
| --- | --- | --- | --- | --- | --- |
| *Murdochiella* | 57255520 | 13723677 | 4.172 | < 0.001 | 15% |
| *Tannerella* | 14553 | 4359 | 3.339 | < 0.001 | 74% |
| *Histophilus* | 9559040 | 3223189 | 2.966 | 0.004 | 10% |
| *Fusobacterium* | 766 | 325 | 2.355 | 0.020 | 93% |
| *Clostridioides* | -3686848 | 1298225 | -2.840 | 0.005 | 24% |
| *Pseudoramibacter* | -196750 | 99363 | -1.980 | 0.050 | 32% |
| *Peptostreptococcus* | 16827 | 6210 | 2.710 | 0.008 | 72% |
| *Haemophilus* | -1382 | 685 | -2.139 | 0.046 | 85% |

Detectability is defined as relative abundance >0. Only genera where relative abundance was significantly associated with overall survival time and was >0 in >10% of patients (as less prevalence was deemed to be clinically irrelevant) in this model were carried over for further analysis.

**Supplementary Figures**

# **Supplementary Figure S1. Distribution of bacterial genera across the cancer genome atlas cohort.**


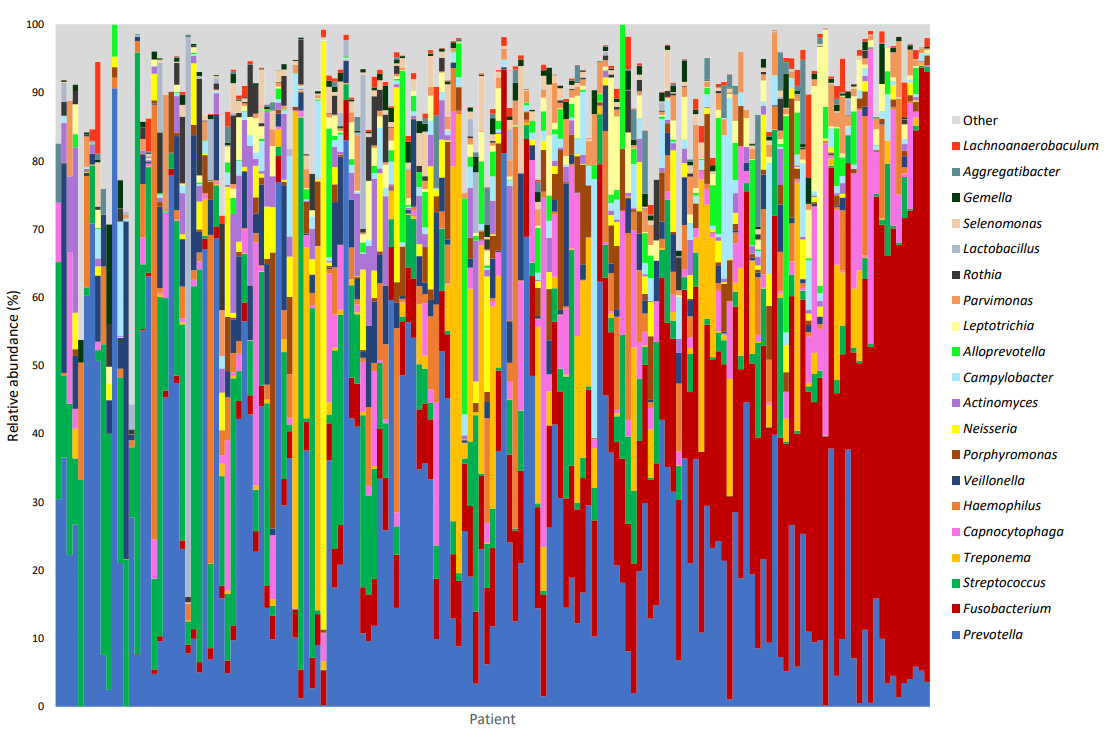


# **Supplementary Figure S2. *Fusobacterium* detectability is associated with better overall and disease-specific survival. This relationship is observed through a multivariate Cox proportional hazards model, which accounts for various factors, specifically other significant bacteria identified through a prior stepwise regression model as described in the Supplementary Text.**


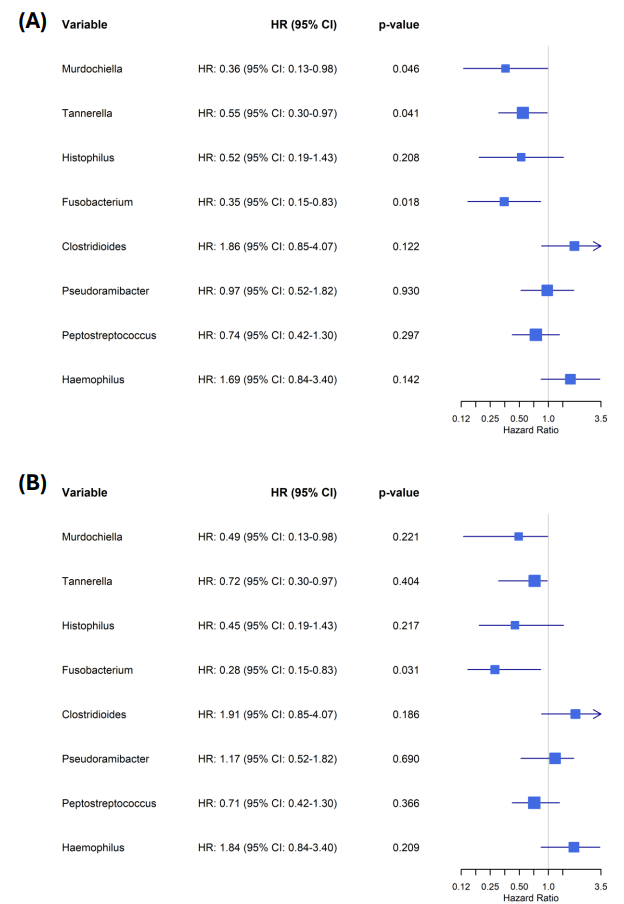


Forest plots with overall (A) and disease-specific survival (B) as the dependent variable.

# **Supplementary Figure S3. *Fusobacterium* is more abundant in HN-SCC compared to normal tissue, and this is not a feature of all Gram-negative commensal oral anaerobic bacteria such as *Prevotella*, which is herein used as a comparator.**


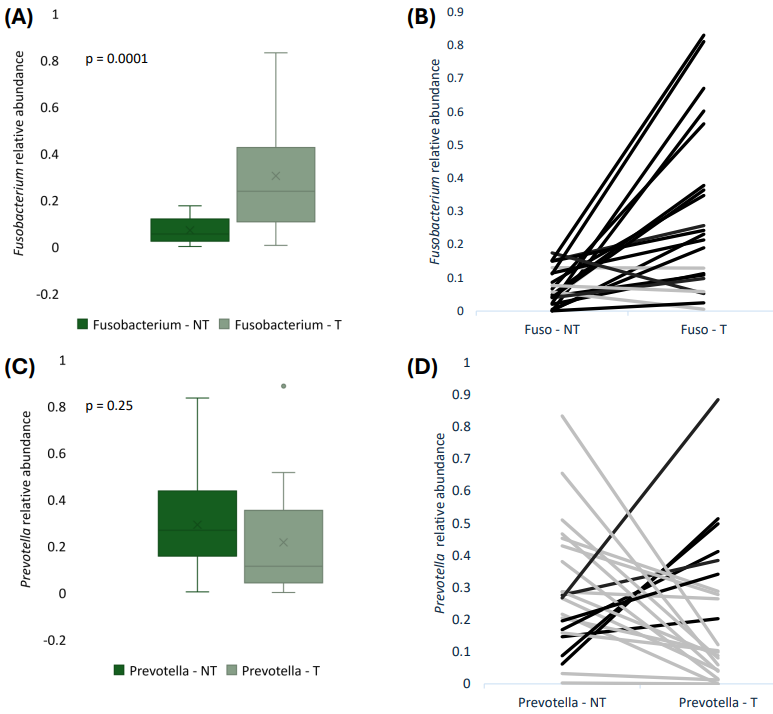


Y axes show relative abundance. Only patients where matched tumor and normal tissue data were available were included (*n* = 21). Left panels: Relative abundance of *Fusobacterium* (A) and *Prevotella* (C) in HN-SCC (T) and matched NT. Right panels: Line plots represent matched samples from each patient, with black and grey lines showing respectively patients with higher and lower relative abundance of *Fusobacterium* (B) and *Prevotella* (D) in tumor compared to normal tissue. These plots show that the relationships suggested by box and whiskers plots are representative of the majority of patients.

Abbreviations: HN-SCC: head and neck squamous cell carcinoma. T: Tumor. NT: Normal tissue.

#

# **Supplementary Figure S4. A higher relative abundance of *Fusobacterium* is associated with survival in the TCMA cohort.**


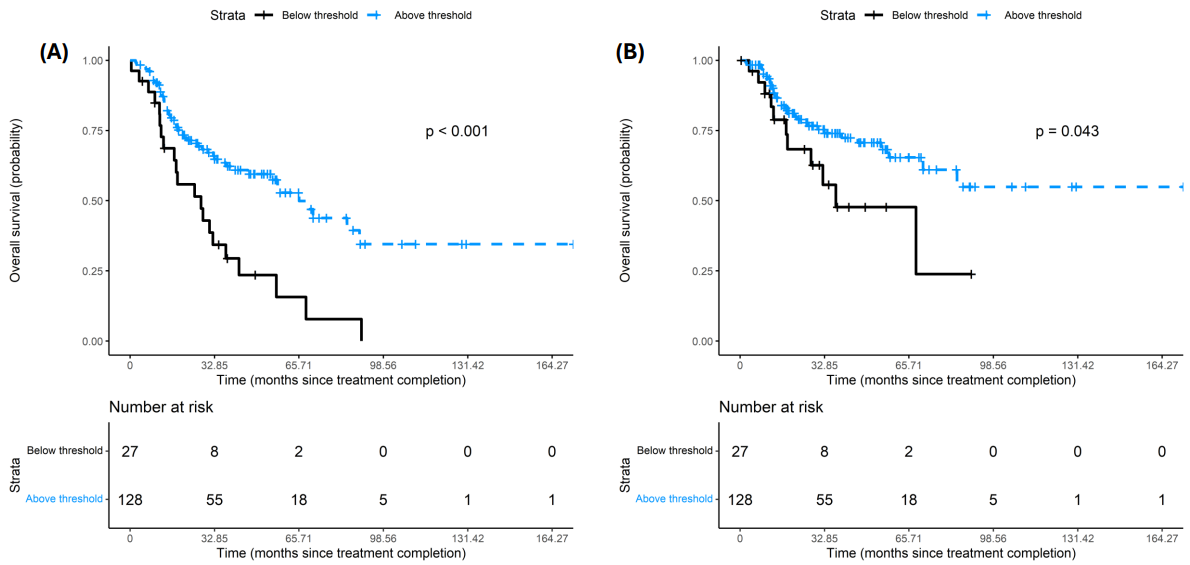


Overall (A) and disease-specific (B) survival in cohort stratified by relative abundance of *Fusobacterium*. Patients were stratified by a cut-off (threshold) defined by ROC analysis as described in the text into those above and below the *Fusobacterium* relative abundance threshold.

Abbreviations: ROC: Receiver Operating Characteristic

# **Supplementary Figure S5. Proportion of patients with detectable *Fusobacterium* species.**


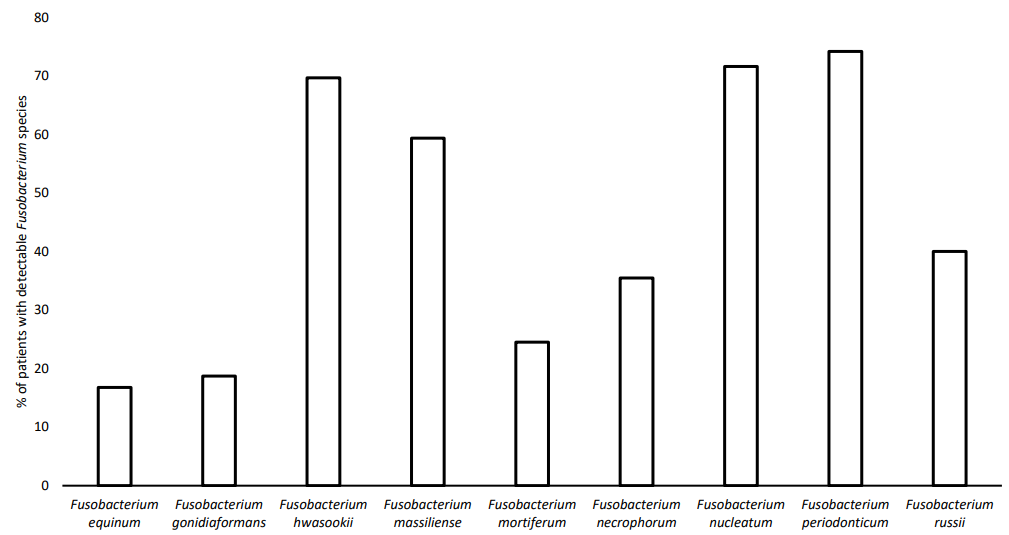


# **Supplementary Figure S6. Cox regression showing that intra-tumoral *Fusobacterium nucleatum* detectability is associated with improved survival among fusobacterial species.**


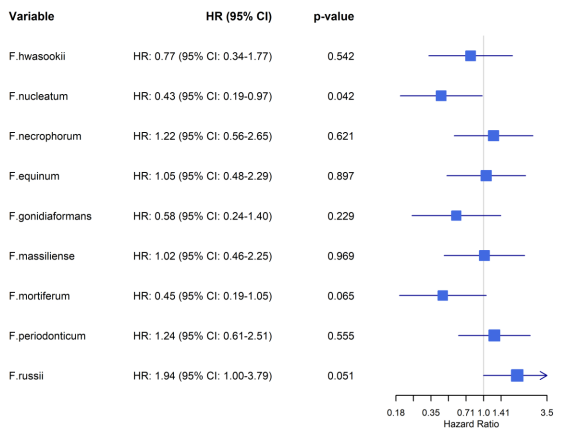


Forest plot of Cox proportional hazards model with overall survival as a dependent variable.

# **Supplementary Figure S7. Intratumoral *Fusobacterium nucleatum* detectability is associated with improved survival in Kaplan-Meier survival analysis.**


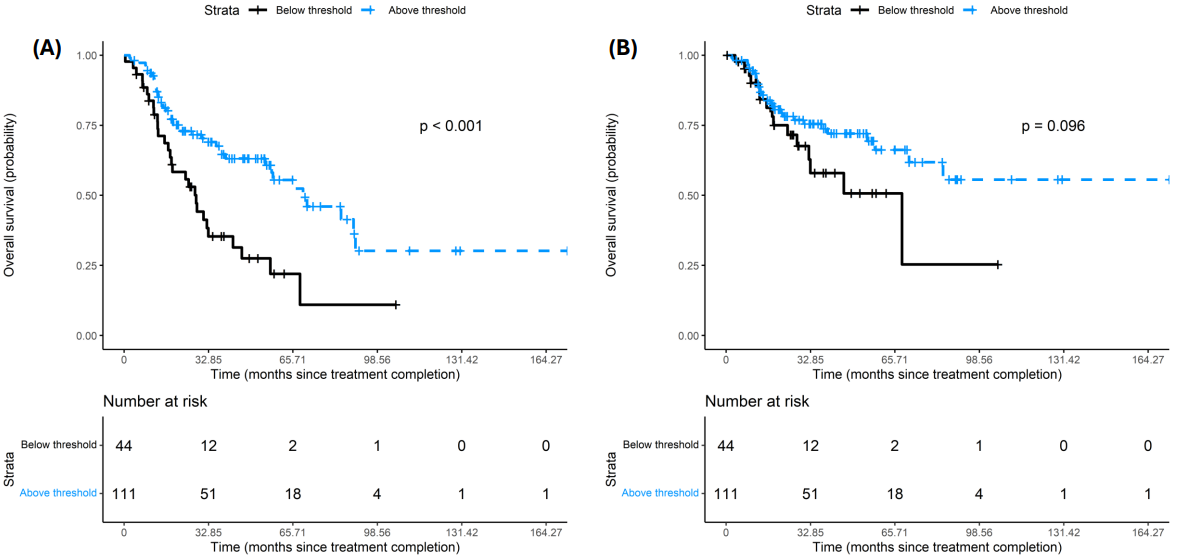


Overall (A) and disease-specific (B) survival in cohort stratified by detectability of *Fusobacterium nucleatum*.

# **Supplementary Figure S8. *Fusobacterium* detectability is significantly associated with better survival when controlling for established predictors of survival.**


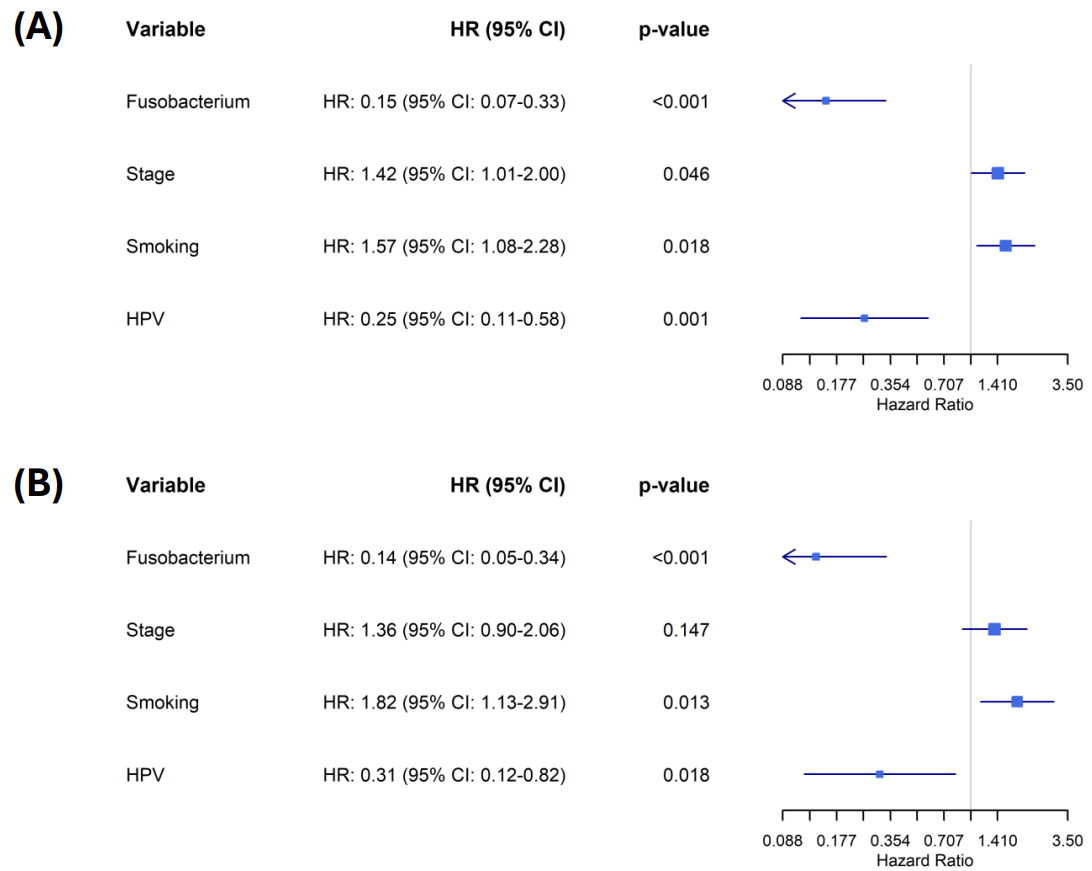


Forest plots with overall (A) and disease-specific (B) survival as the dependent variable. Stage groupings are defined according to the TNM 7th edition for head and neck cancer.

Abbreviations: HPV: Human papillomavirus.

# **Supplementary Figure S9. *Fusobacterium nucleatum* detectability is significantly associated with better survival when controlling for established predictors of survival.**


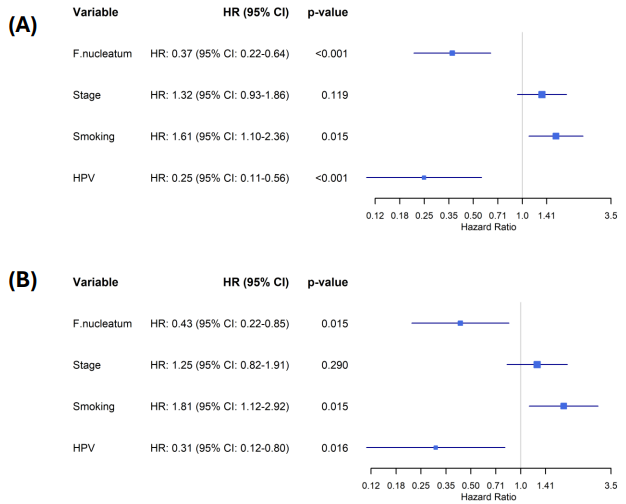


Forest plots with overall (A) and disease-specific (B) survival as the dependent variable. Stage groupings are defined according to the TNM 7th edition for head and neck cancer.

Abbreviations: HPV: Human papillomavirus.

# **Supplementary Figure S10. Patients in the MicroLearner cohort with salivary *Fusobacterium* relative abundance above the cohort median have a better survival.**


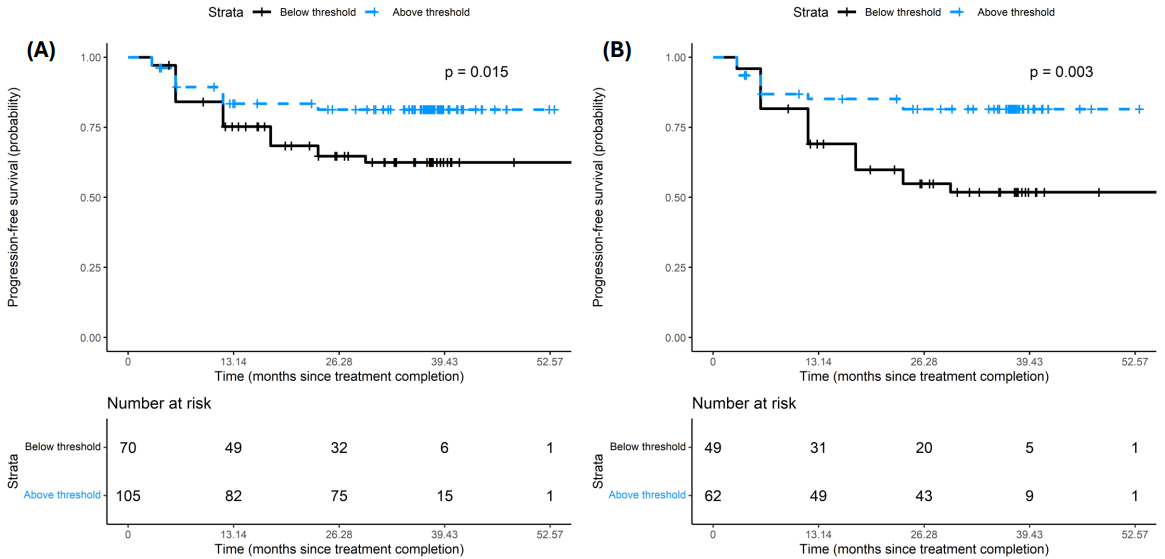


Patients were stratified by a cut-off (threshold) defined by ROC analysis as described in the text into those above and below the *Fusobacterium* relative abundance threshold. **(A)** PFS in the full MicroLearner cohort stratified into groups above and below the threshold relative abundance of *Fusobacterium*. **(B)** PFS in the HPVneg cohort stratified into groups above and below the threshold relative abundance of *Fusobacterium*.

Abbreviations: PFS: Progression-free survival. HPVneg cohort: sub-cohort of patients recruited in the MicroLearner trial with HPV negative head and neck squamous cell carcinoma. *Fnuc*: *Fusobacterium nucleatum*. *Fper*: *Fusobacterium periodonticum*.

# **Supplementary Figure S11. Progression-free survival in the MicroLearner cohort with groups stratified by median *Fnuc* and *Fper*.**


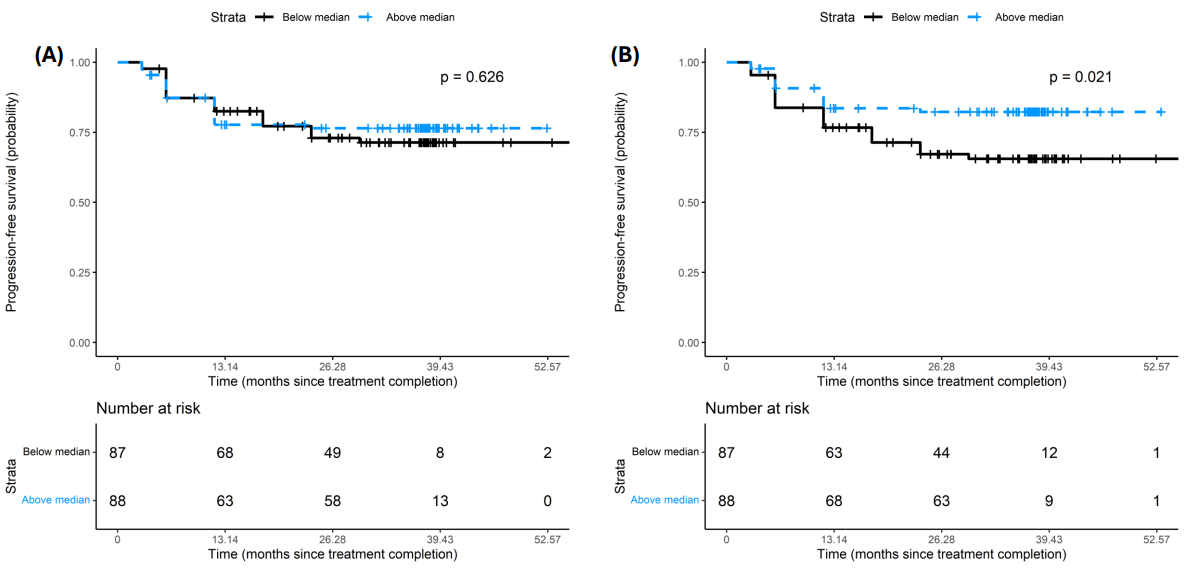


**(A)** PFS in the HPVneg cohort stratified into groups above and below median relative abundance of *F. nucleatum*. **(B)** PFS in the HPVneg cohort stratified into groups above and below median relative abundance of *F. periodonticum*.

Abbreviations: PFS: Progression-free survival. HPVneg cohort: sub-cohort of patients recruited in the MicroLearner trial with HPV-negative head and neck squamous cell carcinoma. *Fnuc*: *Fusobacterium nucleatum*. *Fper*: *Fusobacterium periodonticum*. FnucHI: group of patients above median relative abundance of *F. nucleatum*. FnucLO: group of patients below median relative abundance of *F. nucleatum*. FperHI: group of patients above median relative abundance of *F. periodonticum*. FperLO: group of patients below median relative abundance of *F. periodonticum*.

# **Supplementary Figure S12. *F. nucleatum* causes OSCC killing in 2D cell cultures.**


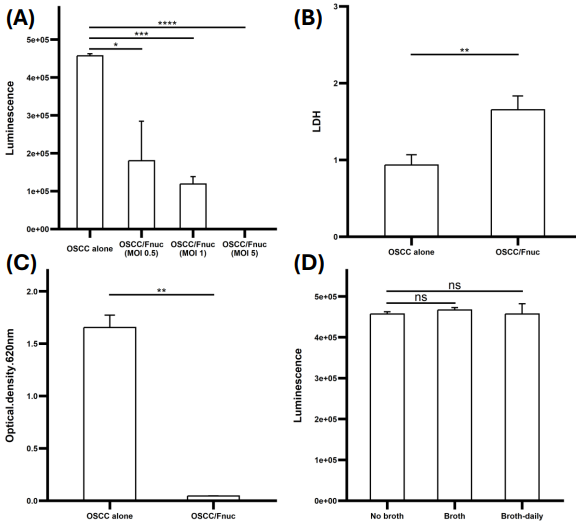


**(A**) OSCC viability as assessed by ATP release (viability measured by luminescence, higher values represent higher viability) at day 5 of co-culture of *F. nucleatum* and OSCC (TR146 cell line) with daily infection. Each bar plot represents the viability of OSCC cultured alone or with *F. nucleatum* at MOI, as indicated in the X-axis. **(B)** OSCC death measured by LDH release (higher values represent more OSCC killing) on day 5 of co-culture. **(C)** OSCC death/viability measured by crystal violet assay (crystal violet staining measured by optical density; higher values indicate more cell viability). **(D)** In this control experiment, adding sterile *F. nucleatum* broth (i.e., native medium - no previous contact with any bacteria) without bacteria to OSCC cell cultures did not have any impact after 5 days with either single ("broth") or daily over 5 days ("broth-daily") addition. All experiments were carried out in triplicate.

Abbreviations: OSCC: oral squamous cell carcinoma (TR146 cell line), Fnuc: *Fusobacterium nucleatum*, MOI: multiplicity of infection, LDH: lactate dehydrogenase. Significance levels: ns: not significant; *:*P* < 0.05; **: *P* < 0.01; ***: *P <* 0.001; ****: *P <* 0.0001.

# **Supplementary Figure S13. OSCC viability decreases over time after infection with *Fusobacterium nucleatum* (MOI 10).**


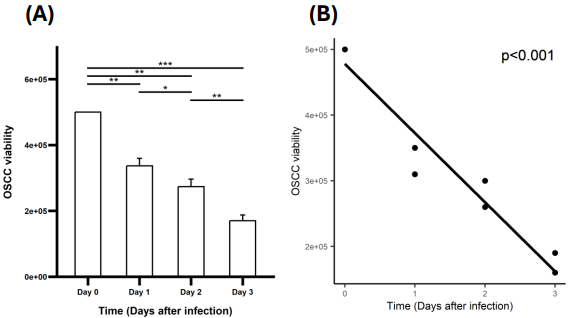


**(A)** Bar charts show the viability of OSCC in the culture the day after infection. **(B)** Linear regression plot of decrease in viability over time. Effects are visible from day 1 after infection.

Abbreviations: OSCC: Oral squamous cell carcinoma. Significance levels: ns: not significant; *: *P <* 0.05; **: *P <* 0.01; ***: *P <* 0.001; ****: *P <* 0.0001.

# **Supplementary Figure S14. OSCC killing (crystal violet assay) in a single culture or in co-culture with either *F. nucleatum* or *P. oralis* (single infection).**


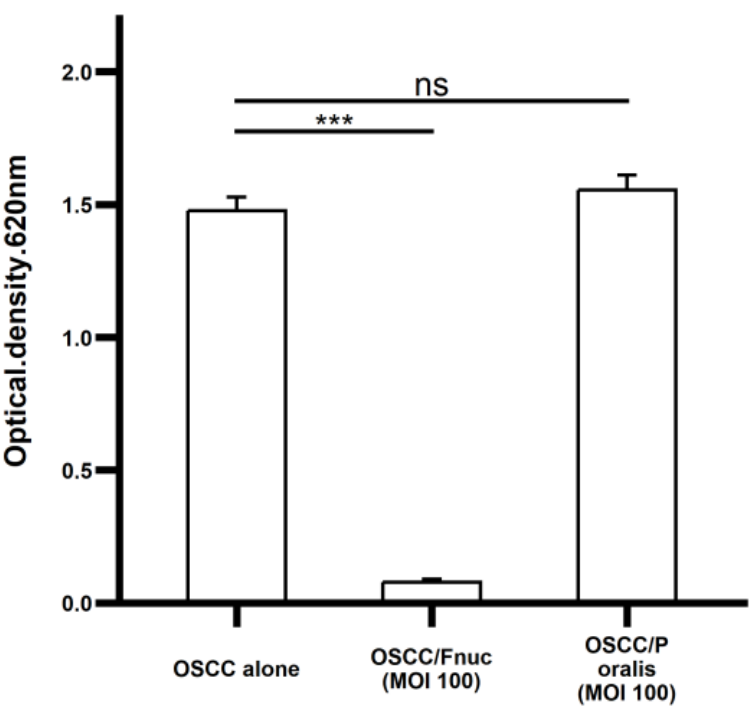


Co-culture of OSCC with *P. oralis* at large MOI (100) does not cause OSCC decreased viability or cell death, whereas *F. nucleatum* (MOI 100) is reproducibly cytotoxic for OSCC in co-culture. Abbreviations:

Abbreviations: OSCC: Oral squamous cell carcinoma, MOI: Multiplicity of infection, *P oralis*: *Prevotella oralis* (NCTC 11459), *Fnuc*: *Fusobacterium nucleatum*. Significance levels: ns: not significant; *: *P <* 0.05; **: *P <* 0.01; ***: *P <* 0.001; ****: *P <* 0.0001.

# **Supplementary Figure S15. *F. periodonticum* and *F. nucleatum* cause OSCC killing analogously at low MOI.**


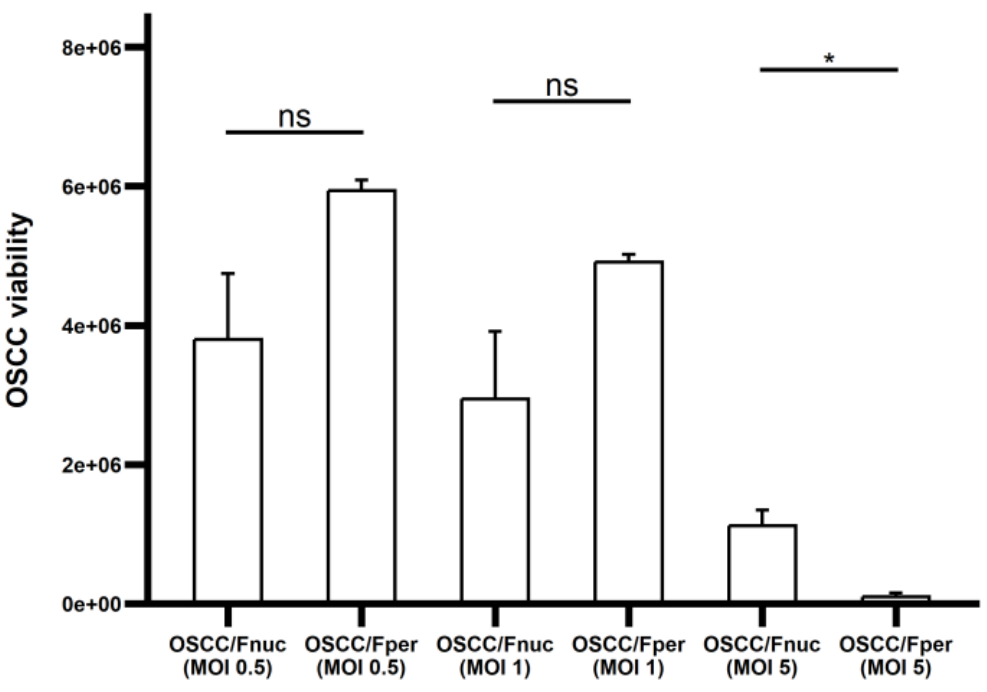


The X-axis indicates the conditions. The viability assay was performed at 5 days after infection.

Abbreviations: OSCC: Oral squamous cell carcinoma. *P oralis*: *Prevotella oralis* (NCTC 11459), *Fnuc*: *Fusobacterium nucleatum*; *Fper*: *Fusobacterium periodonticum*. Significance levels: ns: not significant; *: *P <* 0.05; **: *P <* 0.01; ***: *P <* 0.001; ****: *P <* 0.0001.

# **Supplementary Figure S16. OSCC killing is caused by the fusobacterial secretome (see also Figure 1G).**


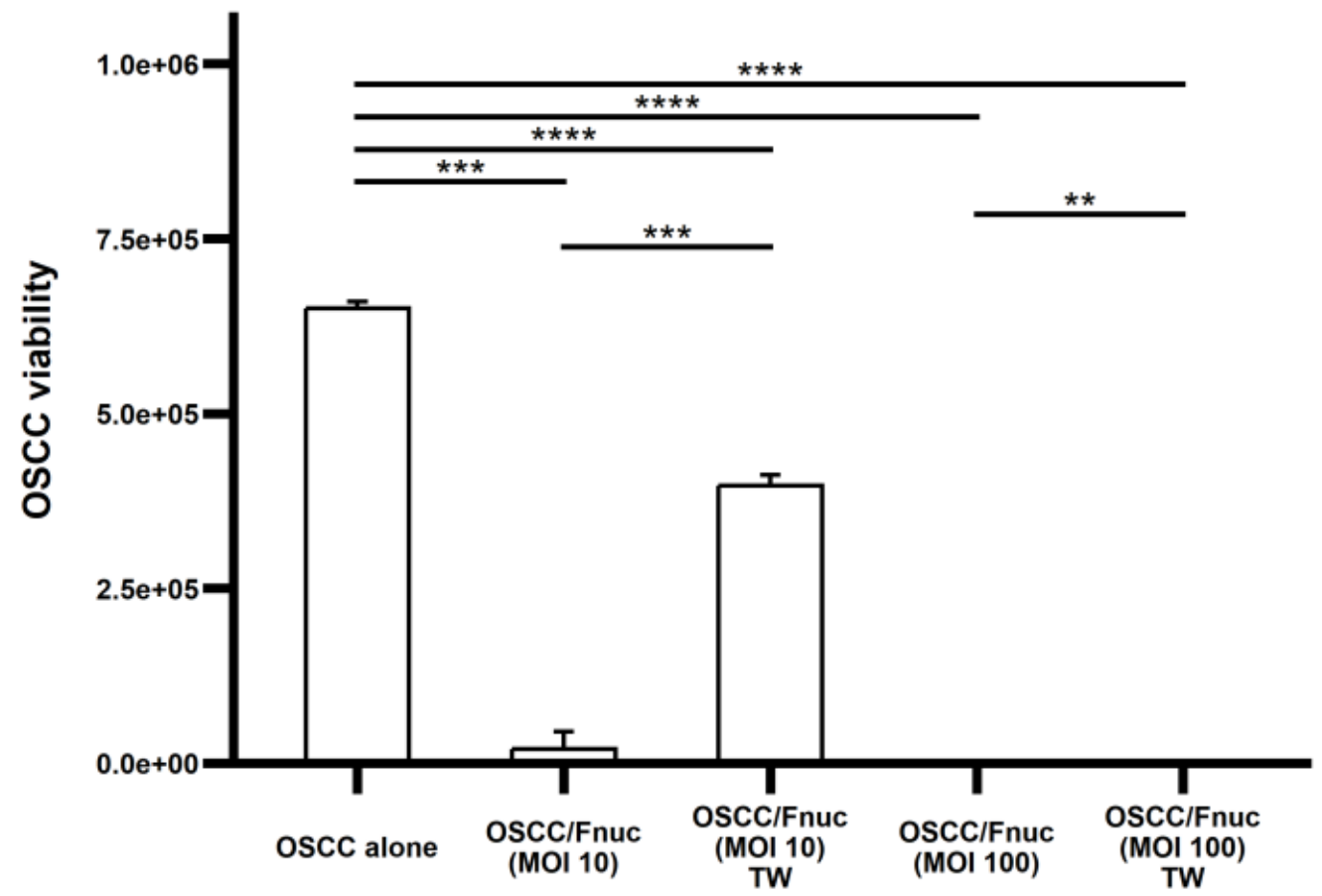


Isolating *Fnuc* (MOI 10 and 100) from OSCC using transwell inserts (TW, which allow the free flow of supernatant molecules but impede bacteria-OSCC contact) decreases OSCC killing significantly. Note: although difficult to see graphically due to the scale of comparison with the control, at MOI 100, mean (SD) OSCC luminescence was 1.3.10^2^ (10.4) vs 1.14.10^3^ (178.2) without and with TW, respectively. All experiments were carried out in triplicate.

Abbreviations: OSCC: Oral squamous cell carcinoma, *Fnuc*: *Fusobacterium nucleatum*, TW: transwell insert used, MOI: Multiplicity of infection, SD: standard deviation. Significance levels: ns: not significant; *: *P <* 0.05; **: *P <* 0.01; ***: *P <* 0.001; ****: *P <* 0.0001.

#

# **Supplementary Figure S17. 2D co-culture experiments timeline.**


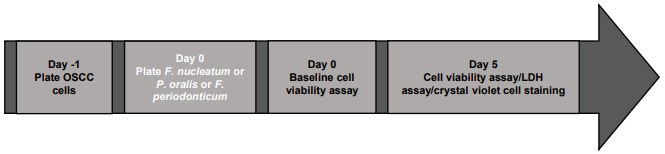


Abbreviations: 2D: two-dimensional co-culture; LDH: lactate dehydrogenase.
